# Rapid Workforce Development to Combat the COVID-19 Pandemic: Experience From a Tertiary Health Care Centre in North India

**DOI:** 10.7759/cureus.15585

**Published:** 2021-06-11

**Authors:** Shalinee Rao, Kusum K Rohilla, Rajesh Kathrotia, Manisha Naithani, Arun Varghese, Anupama Bahadur, Puneet Dhar, Pradeep Aggarwal, Manoj Gupta, Ravi Kant

**Affiliations:** 1 Pathology, All India Institute of Medical Sciences Rishikesh, Rishikesh, IND; 2 Advanced Center of Continuous Professional Development, All India Institute of Medical Sciences Rishikesh, Rishikesh, IND; 3 College of Nursing, All India Institute of Medical Sciences Rishikesh, Rishikesh, IND; 4 Physiology, All India Institute of Medical Sciences Rishikesh, Rishikesh, IND; 5 Biochemistry, All India Institute of Medical Sciences Rishikesh, Rishikesh, IND; 6 Obstetrics and Gynecology, All India Institute of Medical Sciences Rishikesh, Rishikesh, IND; 7 Surgical Gastroenterology, All India Institute of Medical Sciences Rishikesh, Rishikesh, IND; 8 Community & Family Medicine, All India Institute of Medical Sciences Rishikesh, Rishikesh, IND; 9 Radiation Oncology, All India Institute of Medical Sciences Rishikesh, Rishikesh, IND; 10 Director, All India Institute of Medical Sciences Rishikesh, Rishikesh, IND

**Keywords:** covid -19, health care workers, preparedness training, fast track, workforce

## Abstract

Introduction

During a large-scale disease outbreak, one needs to respond to the situation quickly towards capacity building, by identifying areas that require training and planning a workable strategy and implementing it. There are limited studies focused on fast-track workforce creation under challenging circumstances that demand mandatory social distancing and discouragement of gatherings. This study was conducted to analyze the planning process and implementation of fast-track training during the Coronavirus disease (COVID-19) pandemic, and evaluate its effectiveness in building a rapid, skilled, and massive workforce.

Methods

A cross-sectional study was conducted to evaluate rapid preparedness training delivered from March to June 2020, based on documents and data regarding the process, planning, and implementation for large-scale capacity building. Pre-test and post-test scores were compared to assess the effectiveness of training. The number of personnel trained was evaluated to determine the efficiency of the training program. Data on COVID-19 among health care workers (HCWs) were analyzed.

Results

The Advanced Center of Continuous Professional Development acted as the central facility, quickly responding to the situation. A total of 327 training sessions were conducted, including 76 online sessions with 153 instructors. The capacity-building of 2,706 individuals (913 clinicians and 1,793 nurses, paramedics, and non-medical staff) was achieved through multiple parallel sessions on general precautionary measures and specialized skills within four months. The rate of hospital staff infected with COVID-19 was found to be 0.01% over five months.

Conclusions

A fast-track, efficient, large-scale workforce can be created through a central facility even under challenging circumstances which restrict gatherings and require physical distancing. A training action plan for disease outbreaks would be a useful resource to tackle such medical emergencies affecting substantial populations in future.

## Introduction

The rapid and logarithmic spread of the worst pandemic of this century due to the novel Coronavirus disease (COVID-19) has challenged the entire world [1]. One of the main fears was that it would quickly overwhelm healthcare systems. Therefore, to halt the exponential onslaught of the COVID-19 virus, global health systems need to be prepared adequately and appropriately. Apart from slowing the transition from one level to another, preparedness includes ensuring the availability of material resources such as infrastructure, medical equipment and personal protective items for hospitals and optimizing manpower resources like health care workers (HCWs) and other non-medical support personnel. For fulfillment of the last requirement, an important task would be to strengthen and build the capacity of the workforces. Identification of areas requiring training or skill enhancement may differ according to job profiles and roles of HCWs. Intervention processes must be dynamic to keep pace with rapid changes in understanding and management of the novel disease.

Many countries, including India, have implemented lockdowns to stop, contain, control, delay, and diminish the spread of virus by attempting to reduce social contact [2]. Lockdown in itself cannot halt the disease, yet it is invaluable in buying time for the health care system to prepare adequately in terms of resources and strengthen and train manpower efficiently to combat this disease [3,4].

There are limited studies focused on sharing experiences towards fast-track health care professional workforce generation under circumstances demanding mandatory social distancing of a limited duration to combat a highly infectious disease [5-7]. The importance of vigorous preparedness actions responding through a well-coordinated process was highlighted during the Ebola virus disease (EVD) outbreak in 2014-2016 [8]. There was, however, a steep rise in cases towards the beginning of the COVID-19 outbreak, which was attributed to a delayed initial response, resulting in spread of infection among HCWs [9]. Hence, there was a need for rapid dissemination of information on measures that would facilitate preparation of efficient workforces within a short span of time in severely restricted environments. This can even be replicated as and when required in future similar crises.

This study was undertaken to evaluate the strategies and steps taken towards rapid workforce development during the initial phase of the COVID-19 pandemic and determine its effectiveness based on pre-test and post-test scores obtained and also to assess efficiency/productivity of training on the basis of capacity-building and the impact of training programs based on infection rates in HCWs.

## Materials and methods

**Figure 1:**
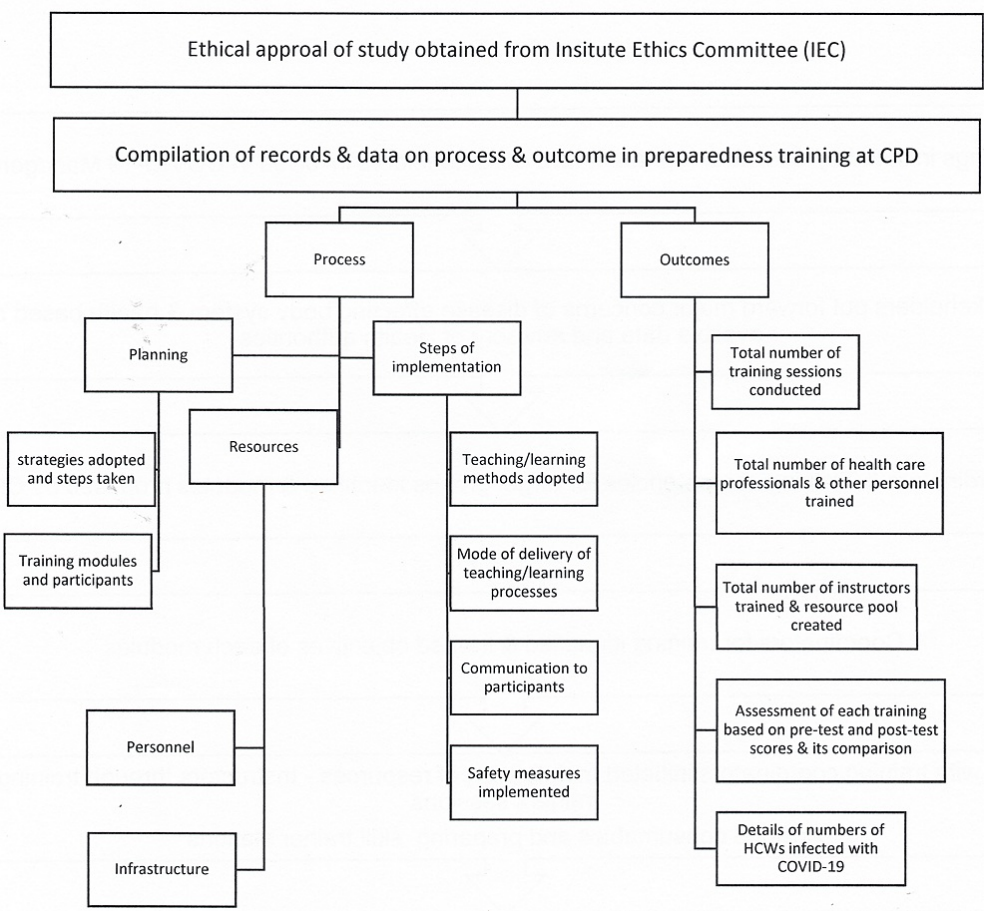
Outline of Methodology

This was a cross-sectional study conducted in the Department of Advanced Center of Continuous Professional Development (CPD) at an apex tertiary care hospital in Uttarakhand, India on the process and output of preparedness training program to combat COVID-19 (Figure 1).

The data obtained during the delivery of preparedness training to combat COVID-19 to all HCWs (Table 1) from March 2020 to June 2020 were included in the study for analysis. The following details were recorded from training data which was used for analysis:

(1) Data regarding processes and strategies followed in planning and steps taken for implementation of proposed training activities.

(2) Total number of health care professionals and other personnel trained.

(3) Total number of instructors engaged, and sessions conducted during training.

(4) Teaching/learning methods adopted and means of delivery of teaching/learning processes.

(5) Assessment of all training based on pre-test and post-test scores and its comparison.

(6) Details of numbers of HCWs infected with COVID-19.

**Table 1:** Preparedness training to combat COVID-19 (March to June 2020) *Safety precautions include Training on General instructions, PPE Donning and Doffing and Hand hygiene; Biomedical Waste Management; Contact Tracing; Cleaning and Disinfection; and Dos and Don’ts. MBBS – Bachelor Of Medicine And Bachelor Of Surgery; BSc – Bachelor Of Science; MSc – Master Of Science; PHC – Primary Health Center MSW – Medical Social Worker; MRC – Medical Record Clerk; BMW – Biomedical Waste Management; PhD- Doctor Of Philosophy; AYUSH – Ayurveda, Yoga and Naturopathy, Unani, Siddha and Homeopathy; OT – Operating Theatre; ECG – Electrocardiography; PPE – Personal Protective Equipment; CPR – Cardiopulmonary Resuscitation; AHA – The American Heart Association. On-site audits were done randomly to monitor compliance. However, no data were available on this.

Session-wise training activities	Target participants	
Training on Airway Management (in COVID-19 Cases)	Faculty, Residents, Nurses, and OT Technicians	
Hand Hygiene	Office Staff, Security Guards, and Housekeeping staff	
*Safety precautions & CPR Recommendations (as per AHA for COVID-19)	Faculty, Residents, Nurses, PhD Scholars, Medical Officers, Tutors, MBBS Students, AYUSH Officers, Interns, Technicians, MSc & BSc Students (Nurses), Technicians, Lab Attendants, Ambulance Drivers (AIIMS and Outside Ambulance Drivers), Medical Social Workers, Pharmacists, PHC Staff	
Sample collection	Faculty, Residents, Nurses, Interns, Lab Technicians	
*Safety precautions	Housekeeping Staff and Hospital Attendants, Housekeeping Supervisors, Sanitation Inspectors, Sanitation Officers and BMW Supervisors, Kitchen Staff, Dieticians, Non-Medical Office Staff, Hostel Wardens, Storekeepers, Security Officers	
Airway Management (in COVID 19-Cases)	Faculty, Residents, Nurses, Pulmonary Medicine Technicians	
Training on Ventilatory Management in Critically ill Patients & Adjuncts	Faculty and Residents	
		Training on ECG Identification of Rhythm & Clinical Management (in COVID-19 Cases)	Faculty and Residents	
Training on Basic Critical Care Skill and Ventilator Support	Nurses	
*Safety precautions ; precautionary measures precooking, cooking and serving food	Hospital Kitchen Staff, Hostel Mess Staff, Canteen Staff & Guest House Staff	
*Safety precautions; Contact Tracing; Cleaning and Disinfection	Nurses, MSWs, MRCs, Drivers, Hospital Attendants, Housekeeping Staff, Ambulance Drivers, Housekeeping Supervisors, Sanitation Inspectors, Sanitation Officers	
Training on Ambulance Disinfection and Handling & Management of Cadavers	Housekeeping Staff, Ambulance Drivers, Housekeeping Supervisors	
Reinforcement Training for Cleaning and Disinfection	Housekeeping Staff, Housekeeping Supervisors, Porters, Sanitation Inspector and Sanitation Officers, BMW Supervisors	

The data of preparedness training to combat COVID-19 during the study period were entered into a Microsoft 2019 Excel spreadsheet, compiled, and analyzed using statistical software IBM SPSS version 23 (IBM Corp., Armonk, NY, USA) for windows. Mean pre-test and post-test scores were compared using paired t test to assess the improvement in knowledge/skills (p values of <0.05 was considered statistically significant).

## Results

Results of the study have been categorized as process and outcome of preparedness training for combating COVID-19 infection.

Process

(i) *Strategies adopted and processes followed to deliver preparedness training for combating COVID-19 infection*

**Figure 2:**
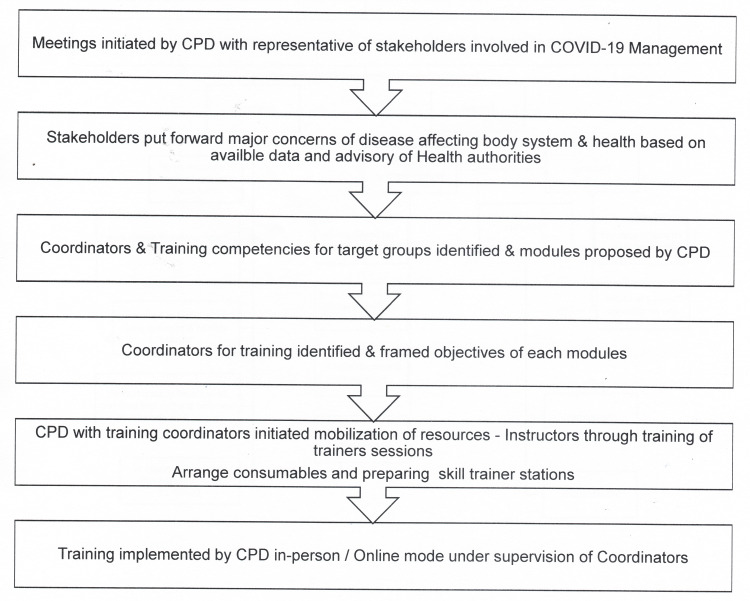
Flowchart depicting sequence of events followed by CPD to conceptualize and implement preparedness training program CPD: Advanced Center of Continuous Professional Development

A centralized approach was followed through the CPD for providing training during the COVID-19 pandemic.

(ii) *Steps followed by the CPD to initiate preparedness training* (Figure 2)

Step 1: Meetings initiated by the CPD with representatives of stakeholders involved in COVID-19 management.

Step 2: Stakeholders put forward major concerns of diseases affecting body systems and health based on the available data and advice of health authorities.

Step 3: Coordinators were identified by the CPD.

Step 4: Target groups were identified, training competencies were specialized, and general (non-specialized) modules were finalized by the CPD with the help of coordinators.

Step 5: Objectives and assessment tools for each training modules were finalized.

Step 6: Mobilization of resources:

(a) Arrangement of all consumables and preparation of skill-training stations was done by coordinators with the support of the CPD.

(b) Mobilization of instructors by conducting training of trainers (TOT) sessions before starting training sessions for all HCWs.

Step 7: Communication about the training was sent by the CPD to participants and the officer-in-charge by email.

Step 8: Training for HCW implemented by the CPD in-person/online mode under the supervision of coordinators.

(iii) *Training modules*

Based on major health-related concerns in patient care of COVID-19 cases and opinions of Experts and Advisory released by the Ministry of Health and Family Welfare (Government), the World Health Organization (WHO), and the American Heart Association (AHA), training modules were directed towards the acquisition of competencies specific for the direct and indirect patient-caregiver of hospitals (Table 1). Competencies that were related to each other were combined and delivered together in one session to avoid participants attending training multiple times (Table 1).

(iv) *Resources*

Planning was done to mobilize resources (Table 2) for training (instructors and infrastructure).

▪ Personnel

To prepare a pool of instructors, selected health personnel underwent TOT for two days before initiating training of all health care professionals.

Implementation of training modules was carried out with an appropriate instructor-to-participant ratio and as small group sessions (Table 2).

▪ Infrastructure - Places, methods and modes of training

(a) Venues included multiple halls, auditoriums, large open corridors, cul-de-sacs and simulation labs in order to run parallel training sessions at the same time. Each venue was equipped with the proper audio-video aids and seating arrangements. Limited numbers of participants (Table 2) were present at each venue to maintain physical distancing. Parallel multiple sessions were conducted six days a week to expedite training.

(b) From the month of May (coinciding with a sudden increase of cases in the state), online training was provided through the Google Meet platform and meeting links were shared with participants. Good bandwidth connectivity was essential for uninterrupted and good quality video and audio, which was ensured by information and technology (IT). Some sessions were delivered by instructors from the CPD hall (where props were essential), and some were delivered from their respective locations/departments. Participants were instructed to be available with a laptop and check for good Wi-Fi connectivity prior to the sessions.

**Table 2:** Resources in terms of manpower and equipment/consumables to facilitate preparedness training to combat COVID-19 *Safety precautions include training on general instructions, PPE donning and doffing and hand hygiene, biomedical waste management, contact tracing, cleaning and disinfection, and dos and don’ts. ECG – Electrocardiography; PPE – Personal Protective Equipment; CPR – Cardiopulmonary Resuscitation; AHA – The American Heart Association

Competencies	Number of instructors and participants per session	Equipment/consumables	Teaching Learning Method
Hand Hygiene	4 per 25-30	Hand sanitizer	Demonstration and hands-on training
Hand Hygiene and PPE Donning and Doffing	4 per 25-30	PPE kit and hand sanitizer for demonstration	Demonstration and interactive small group discussion
*Safety precautions; Sample collection; Contact Tracing	4 per 25-30	Laptop; projector and screen video demonstration	Video demonstration and interactive small group discussion
CPR Recommendations (as per AHA for COVID-19)	1 per 6	Half-body mannequin for CPR and automated external defibrillator (AED)	Demonstration and hands-on training on low-fidelity simulator
Airway Management (in COVID-19 Cases)	4 per 20	Video demonstration, PPE kit, hand sanitizer, airway task trainer and adjuncts, high-fidelity human patient simulator	Demonstration and hands on training on task trainer and high-fidelity simulator
Ventilatory Management in Critically ill Patients and Adjuncts	4 per 20	High-fidelity human patient simulator, ventilator, task trainer for arterial puncture and suction, video demonstration, hand sanitizer	Demonstration and hands on training on task trainer and high-fidelity simulator
ECG Identification of Rhythm and Clinical Management (in COVID-19 Cases)	1 per 20	Laptop and projector and screen	PowerPoint presentation and interactive small group discussion
Basic Critical Care Skill and Ventilator Support for Nurses Officers	5 per 25	Ventilator, task trainer for arterial puncture and suction, oral feeding through tubes, electrocardiogram machine, video demonstration, hand sanitizer	Demonstration and hands on training on task trainers
Precautionary measures precooking, cooking and serving food	4 per 20	Hand sanitizer, laptop and projector and screen	Video demonstration and interactive small-group discussion
Quarantine Standard Operating Procedures (SOP)	2 per 10	PowerPoint display laptop and projector and screen	PowerPoint presentation and interactive small-group discussion
Ambulance Disinfection	2 per 10	Laptop and projector and screen	PowerPoint presentation and interactive small-group discussion
Management and handling of Cadavers	2 per 10	Hands-on training in mortuary with non-COVID-19 cadavers	Demonstration and hands-on training

(v) *Communication to participants*

The CPD informed participants (clinicians and nurses individually) about the training date and time through e-mail one day prior to training. For the non-clinicians, their overall administrative in-charge was the point of contact and the participant list was shared with them through email, a day prior to training. A priority list for order of training of personnel groups was coordinated with the duty rostering administration to ensure training of personnel before they were rostered, as well as to prevent clashes between training and deployment.

(vi) *Safety measures implemented before and during sessions at all venues*

(a) The CPD ensured adequate hand sanitizer was available in each station and at the entry and exit areas to maintain hand hygiene.

(b) Thermal screening was implemented for all.

(c) Wearing face masks, the use of hand sanitizer, and social distancing were also strictly enforced during training.

Outcomes

(i) *Sessions delivered during preparedness training*

Within four months, 327 sessions were conducted with the help of 153 Instructors for 16 different training sessions (Table 3). Seventy-six sessions were delivered through online mode during this period to maintain strict distancing, in which 2,706 participants were trained by 85 instructors (Table 3).

**Table 3:** Training sessions and number of participants trained from March to June 2020 *Safety precautions include training on general instructions, PPE donning and doffing and hand hygiene, biomedical waste management, contact tracing, cleaning and disinfection, and dos and don’ts. PPE – Personal Protective Equipment; CPR – Cardiopulmonary Resuscitation; AHA – The American Heart Association)

Training Program	Instructors	Number of sessions – In person training	Number of sessions – Online training	Total number of sessions completed in 4 months	Participants trained Online	Total number of Participants trained
Hand Hygiene	16	18	-	18	-	280
Hand Hygiene and PPE Donning and Doffing	4	14	-	14	-	537
*Safety precautions; sample collection; contact tracing	33	46	14	60	422	1703
*Safety precautions; Contact Tracing		34	-	34		1003
CPR Recommendations (as per AHA for COVID-19)	15	46	14	60	422	1703
Airway Management (in COVID-19 Cases)	11	26	17	43	1036	1668
Ventilatory Management in Critically ill Patients and adjuncts	12	6	15	21	457	560
ECG Identification of Rhythm and Clinical Management (in COVID-19 Cases)	14	19	16	35	239	856
Basic Critical Care Skill and Ventilator Support	18	9	-	9	-	287
Video laryngoscopy	4	7	-	7	-	152
Interactive session with security guards to alleviate fear, anxiety, to follow common sense and promote correct practices in COVID-19 pandemic	6	10	-	10	-	269
*Safety precautions; precautionary measures precooking, cooking and serving food	4	2	-	2	-	50
Quarantine Team responsibilities and workflow	4	4	-	4	-	73
Ambulance Disinfection	4	2	-	2	-	30
Handling and Management of cadavers	4	2	-	2	-	30
Reinforcement Training for Cleaning and Disinfection	4	6	-	6	-	140
Modules	153	251	76	327	-	-

(ii) *Modes of assessment followed in training modules*

To assess the effectiveness of each training provided, an objective assessment was followed. A pre-validated questionnaire based on the content delivered specifically to that training was administered to participants through multiple-choice questions in pre-test and post-test by online mode (Google Forms). Achieving a cutoff score of 70% by the participant was required for successful completion of training. Participants who achieved a score below 70% were re-trained.

(a) General module for COVID-19

- Who were trained, capacity building number, and content: Clinicians and non-clinicians (2,706 participants) were trained for general instructions to be followed during COVID-19. It included hand hygiene, general instructions, the donning and doffing of personal protective equipment, cleaning and disinfection, and dos and don’ts. Pre-test and post-test scores analysis showed improvement in knowledge which was found to be statistically significant (Table 4).

(b) Special module for medical clinicians for COVID-19

- Who were trained, their number, and content: A total of 751 clinicians comprised of faculty members, senior residents, junior residents, and interns were trained. The main areas of training were CPR recommendations and airway management for COVID-19 cases, sample collection, contact tracing, ventilator management in critically ill patients and adjuncts, and ECG identification of abnormal rhythms and their clinical management. Pre and post-test assessments were compared (Table 4). Improvement in knowledge was found to be statistically significant (all p < 0.001) among the clinician group.

(c) Special module for medical staff (non-clinicians)

- Who were trained, their number, and content: A total of 1,278 non-clinicians comprised of nurses’ officers, technicians and other paramedical staff of the hospital were trained. The main areas of training were management in critically ill patients and adjuncts, CPR recommendations and airway management for COVID-19 cases, environmental sanitation, handling cadavers, ambulance disinfection, and precautionary measures during preparation, cooking, and serving of food (Table 4). Pre and post-test knowledge were compared and showed improvement in knowledge, which was found to be statistically significant (Table 4).

**Table 4:** Performance of participants based on pre-test and post-test assessment ECG – Electrocardiography, CPR – Cardiopulmonary Resuscitation (min-max pre-test and post-test scores are put up in appendices)

Participant	Skills	Number of Participants	Pre-test (Mean ± SD)	Post-test (Mean ± SD)	p-value
Clinicians	General instructions for prevention of COVID-19	913	10.6 ± 2.6	13.1 ± 1.2	<0.001
Non-clinicians		1793	9.9 ± 1.7	11.7 ± 2.6	<0.001
a. Nurses		881	10.0 ± 2.7	12.1 ± 3.1	<0.001
b. Paramedical & Non-Medical Staff		912	9.8 ± 0.8	11.4 ± 2.4	<0.001
Clinicians	a. CPR recommendations and airway management	751	9.7 ± 2.6	11.6 ± 2.3	< 0.001
	b. Ventilator management in critically ill patients & adjuncts	610	6.1 ± 2.2	11.1 ± 2.1	< 0.001
	c. ECG identification of rhythm & their clinical management	856	6.7 ± 1.9	12.6 ± 1.5	< 0.001
	d. Video laryngoscope	152	6.34±1.84	7.62±1.92	< 0.001
Nurses & paramedics	a. Management of critically ill patients and adjuncts	287	6.0 ± 2.4	11.0 ± 2.2	< 0.001
	b. CPR recommendations and airway management	881	9.2 ± 2.9	11.4 ± 2.1	< 0.001
	c. Environmental sanitation, handling cadavers	30	6.6 ± 2.6	12.1 ± 1.8	< 0.001
	d. Ambulance disinfection	30	7.7 ± 2.9	13.6 ± 1.9	< 0.001
	e. Precautionary measures during precooking, cooking & serving food	50	7.1 ± 2.4	13.4 ± 1.8	< 0.001

(iii) *Institute staff infected with COVID-19 from March 2020 to July 2020*

Out of the 3,408 participants who underwent training in the institute, 38 individuals became infected (Table 5). The percentage of HCW /non-medical staff infected with COVID-19 from March to July 2020 was found to be 0.01%. A regular audit on HCW infected with COVID-19 was conducted with root-cause analysis. Corrective and preventive actions were taken based on the results of the audit, such as instructions to all HCWs that during donning and doffing of PPE a “buddy” should be present.

**Table 5:** Health Care workers and Non-medical staff infected with COVID-19 from March to July 2020 PPE- Personal Protective Equipment

HCWs/non-medical staff	Possible reason for exposure		Total HCWs infected
	In-hospital PPE breach or precautions not taken	Outside hospital	
Nurses	10	8	18
Hospital or Lab Attendants/Housekeeping Staff	3	1	4
Doctors (Resident/Medical Faculty)	5	2	7
Interns	1	1	2
Security Guard/Supervisors	1	2	3
Non- Medical staff	1	1	2
Medical / Nursing Students	0	2	2
Total Number	21	17	38

(iv) *Sessions to manage stress and anxiety during COVID-19*

Security staff had interactive sessions in batches with the Division of Medical Humanities to discuss anxieties, fears, day-to-day problems and methods to overcome these concerns. There was a discussion with security staff on how to apply and follow commonsense procedures during the COVID-19 pandemic, as well as on the dispelling of myths (Table 3). A guest lecture was scheduled for all HCWs through online mode.

## Discussion

The infrastructure of the healthcare system worldwide is facing serious challenges under the novel coronavirus pandemic [10]. The daily increase in caseload led to the exhaustion of resources mainly due to ill-equipped facilities for managing this pandemic [6]. In the present situation posed by COVID-19, HCWs comprise a precious resource for any nation [11]. Brainstorming by subject experts and administrators is pivotal for ensuring the identification of all areas requiring training and for framing their learning objectives. The success of our training may be attributed to comprehensive training modules, which were based on disease biology and implemented by experts and coordinators. For this extremely infectious disease, training covering several aspects was necessary which, comprised hand hygiene practices, biomedical waste management, PPE donning and doffing, airway management and ventilator support. Knowledge regarding AHA recommendations on basic life support (BLS), such as leaving oxygen delivery masks in position during cardiopulmonary resuscitation (CPR) limits the spread of aerosols was imparted [12]. Being infected with COVID-19 per se and drugs in advisory can cause rhythm abnormalities, hence ECG changes and management was discussed [13]. Improvement in the knowledge of participants was statistically significant and reflected the effectiveness of rapid preparedness training at our center. A large number of HCWs trained within a short span of four months stands is as evidence of the efficiency of the training and the effectiveness of the strategies implemented. High and low fidelity simulators and part-task trainers played a vital role in facilitating skills delivery in aerosol-generating procedures as well as in intensive care unit skills enhancement, a finding comparable to others studies [6, 14]. TOT is a pivotal step in such rapid large-scale training to produce adequate instructors, and this was a major contributory factor in the success of our program. The availability of appropriate infrastructures such as training spaces and audio-visual systems to run multiple parallel sessions, as well as manpower resources to train the participants, facilitated the rapid generation of a workforce during the lockdown period.

Any breach in the practice of infection control is a key factor that can cause transmission of pathogenic organisms, which can result in further infection spread [15]. With reference to COVID-19, among HCWs an infection rate of about 9% has been documented in the literature [14]. The low infection rate of COVID-19 among the HCWs at our institute reflects the successful results of our preparedness training. Necessary corrective steps were taken based on the audit report of the HCW infected in our hospital. Similar to a study by Gupta et al., a “buddy system” was re-emphasized during the PPE donning and doffing process to avoid contamination of self and environment, based on the audit report of our institute [15]. An analysis of data on institute staff infected with COVID-19 infection during the study period showed that the infection rate was highest among nurses. However, on doing the contact tracing it was found that the source of infection was not only from within but outside the hospital also. This may possibly be due to the laxity in COVID-19 appropriate behavior by them. It is further emphasized HCWs may not necessarily be infected while working in the hospital. Hence, during training sessions, it should be re-emphasized to HCWs to follow all preventive measures even outside the hospital premises.

It is also important to focus on relieving the anxiety of HCWs and address the burn-out issues of HCWs while dealing with an outbreak/pandemic [16]. In the early phase of the COVID-19 pandemic, our institute had already prioritized skill development to safeguard the health of our HCWs and patient care/management, and therefore sessions on stress management were minimal during this period.

One of the steps before formulating any strategies is the inspection of similar situations, such as the EVD outbreak of 2014 and devising a plan to avoid any lacunae within the existing system. In the EVD emergency of 2014, deficiencies were noted in the prompt support to facilitating capacity-building in the affected areas [17]. Even in the COVID-19 pandemic situation, in the early phase of the pandemic, there was no readily available format of training modules for preparing the workforce. Only advisories released by health authorities based on which training could be administered were available. However, at our institute strategic planning with all stakeholders and training coordinators led to a well-structured training program and the creation of a rapid-response workforce. One highlight of the fast-track training was effective communication and organizing skills delivered through the central unit of the CPD. Implementation measures undertaken by the CPD resulted in good capacity-building overall. This emphasizes the importance of having a central facility to rapidly respond and organize training through an integrated coordinated approach [6]. A central facility ensures no scope of misguidance or lack of communication, which can pose a potential hindrance to achieving successful results in any multidisciplinary activity. Angeloni et al. conducted a study to ascertain the distinct organizational features that influence training as well as to specify barriers and possible ways to improve on it. They found that training can be better facilitated by centralizing it and assigning this responsibility to a specific person [18]. Our study also highlights the importance of CPD, which acted as a fulcrum for planning and execution in this rapid creation of a workforce.

Orda et al. studied the major role played by governance in recruiting and retaining a suitably trained medical workforce to care for rural and remote Australians [19]. In the event of an EVD outbreak, measures were developed and implemented between 2014 and 2017 to strengthen local capacity and combat disease transmission, which included the training of HCWs on treatment, safe and quality services, and a rapid response team [8]. Bemah et al. highlighted that no formal training to combat the Ebola virus infection existed in Liberia before the EVD outbreak. However, as the EVD outbreak intensified in 2014 a serious need for building the capacity and confidence of HCWs was acknowledged. To address these issues, an HCW training program was commenced, and the outcome of this program led to improvement in the knowledge, skill, and confidence of HCWs. The testimony to the success of these programs is that following the initiation of training, infection rates dropped among HCWs and no infections among HCWs were found in the post-EVD period [8].

The existing workforce was overburdened in the early phase of the COVID-19 outbreak due to the alarming rate of spread. To maintain an adequate pool of caregivers, training across specialties was the need of the hour [20]. Our study reveals that fast-track training resulted in good capacity-building, in spite of the restrictive environment required during training due to the highly infectious nature of the virus. Li et al. put forward their experience on training to combat COVID-19 infection, recommending continuous education as a step to tackle future emergencies [21]. Experience during such unprecedented situations helps in developing real-time policies [22,23].

An urgent need for preparedness training was of paramount importance in the combat against COVID -19 to prevent the overwhelming of healthcare systems, especially in resource-constrained countries such as ours [24]. However, the main challenge faced was planning and implementation of training within the stipulated time frame [7]. An action plan successfully executed by us to respond to this emergency affecting large scale populations can be a useful resource and be implemented whenever required to appropriately control a similar situation in future.

The highly infectious nature of this disease calls out for rigorous preparedness to combat it [25]. It has become the responsibility of every health care institute to deliver preparedness training for two simple reasons: (i) to ensure the safety of HCWs and ii) to equip HCWs with the necessary skills to take care of COVID-19 patients. However, preparation in a country with a considerably high population and limited infrastructure in terms of health care facilities is definitely a herculean task [5].

The challenges faced in the implementation of the preparedness training program included the following. (i) Involving many stakeholders as the care of the COVID-19 patient was diverse in nature. (ii) Preparation of multiple training modules at a very short time to cater to variable group based on the level of competency expected out of them. (iii) Conducting the training during the ongoing pandemic situation. (iv) Coordination with roster committee as most of them were already posted in the patient care due to sudden emergency. (v) Availability of instructors as they were required also in-patient care area for their expertise in specialized treatment. Limitation in the implementation of the preparedness training program included change in the mode of training from in-person to online delivery of training when there was a sudden surge in cases in our region and country.

## Conclusions

The period of lockdown during the COVID-19 pandemic was utilized sensibly to strengthen the knowledge and skills of HCWs for COVID-19 patient care. A fast-track, efficient workforce can be created under challenging circumstances which restrict gathering and require physical distancing, through strategic planning and implementation through a central facility. Parallel training sessions and rapid creation of a good pool of instructors facilitates fast-track mass-training activities. The availability of an action plan for training would be a useful resource, one that could be adopted and implemented to rapidly respond to and control medical emergencies affecting populations on a large scale.

## Appendices

**Table 6:** Minimum-maximum scores of participants in pre-test and post-test assessment

Participant	Skills	Number of Participants	Pre-test (score min-max)	Post-test (score min-max)
Clinicians	General instructions for prevention of COVID-19	913	4-14	8-15
Non-clinicians		1793	5-13	6-15
a. Nurses		881	3-14	4-15
b. Paramedical & non-medical staff		912	6-13	6-15
Clinicians	a. CPR recommendations and airway management	751	5-13	7-15
	b. Ventilator management in critically ill patients & adjuncts	610	2-11	5-15
	c. ECG identification of rhythm & their clinical management	856	3-12	6-15
	d. Video laryngoscope	152	2-12	3-13
Nurses & paramedics	a. Management of critically ill patients and adjuncts	287	2-13	5-15
	b. CPR recommendations and airway management	881	3-14	4-15
	c. Environmental sanitation, handling cadavers	30	2-12	5-14
	d. Ambulance disinfection	30	2-13	6-15
	e. Precautionary measures during precooking, cooking & serving food	50	2-12	5-15
